# A Nationwide Survey of Mild Cognitive Impairment and Dementia, Including Very Mild Dementia, in Taiwan

**DOI:** 10.1371/journal.pone.0100303

**Published:** 2014-06-18

**Authors:** Yu Sun, Huey-Jane Lee, Shu-Chien Yang, Ta-Fu Chen, Ker-Neng Lin, Chung-Chih Lin, Pei-Ning Wang, Li-Yu Tang, Ming-Jang Chiu

**Affiliations:** 1 Department of Neurology, En Chu Kong Hospital, New Taipei City, Taiwan; Department of Neurology, National Taiwan University Hospital, College of Medicine, National Taiwan University, Taipei, Taiwan; 2 Taiwan Alzheimer’s Disease Association, Taipei, Taiwan; 3 Department of Neurology, National Taiwan University Hospital, College of Medicine, National Taiwan University, Taipei, Taiwan; 4 Department of Neurology, Neurological Institute, Taipei Veterans General Hospital; Department of Psychology, Soochow University, Taipei, Taiwan; 5 Department of Computer Science and Information Engineering, Chang Gung University, Taoyuan, Taiwan; 6 Department of Neurology, Neurological Institute, Taipei Veterans General Hospital; Department of Neurology, School of Medicine, National Yang-Ming University, Taipei, Taiwan; 7 Department of Neurology, National Taiwan University Hospital, College of Medicine; Graduate Institute of Brain and Mind Sciences; Graduate Institute of Psychology; Graduate Institute of Biomedical Engineering and Bioinformatics; National Taiwan University, Taipei, Taiwan; Cardiff University, United Kingdom

## Abstract

An increasing population of dementia patients produces substantial societal impacts. We assessed the prevalence of mild cognitive impairment (MCI) and all-cause dementia, including very mild dementia (VMD), in Taiwan. In a nationwide population-based cross-sectional survey, participants were selected by computerized random sampling from all 19 Taiwan counties and were enrolled between December 2011 and March 2013. Cases were identified through in-person interviews based on the National Institute on Aging-Alzheimer’s Association clinical criteria. Demographic data and histories involving mental status and function in daily living were collected. The principal objective assessments were the Taiwanese Mental Status Examination and Clinical Dementia Rating. In all, 10,432 people aged 65 years or older (mean age 76.2±6.7, 52.3% women) were interviewed. The age-adjusted prevalence of all-cause dementia was 8.04% (95% CI 7.47–8.61), including a 3.25% (95% CI 2.89–3.61) prevalence of VMD; that of MCI was 18.76% (95% CI 17.91–19.61). Women had a higher prevalence than men of both all-cause dementia (9.71% vs. 6.36%) and MCI (21.63% vs. 15.57%). MCI affects a considerable portion of the population aged 65 and above in Taiwan. The inclusion of VMD yields dementia prevalence rates higher than those previously reported from Taiwan. Old age, female gender, and a low educational level are significant associated factors.

## Introduction

The global prevalence of dementia doubles with every 5-year increase in age after 65 years [1]. The aging population in Taiwan has grown rapidly in the past decades, with an increase in the percentage of the population over age 65 from 6.8% in 1992 to 11.1% in 2012 [2]. The size of the dementia population is expected to increase, with substantial societal impacts on health care costs and caregiving.

Previous to this study, no nationwide prevalence report was available for dementia in Taiwan [3]. In addition, people with mild cognitive impairment (MCI) or very mild dementia (VMD) were not specifically assessed. Estimates of the prevalence of MCI throughout the world have ranged widely among studies [4]–[6]. A limited number of epidemiological studies of VMD are available [7], [8]. MCI, VMD, and dementia of other severity stages have different public health implications with varied prognoses, symptoms, treatments, and cost burdens.

Our study aimed to assess the prevalence of MCI and dementia, including VMD, in a representative sample of people aged 65 and over in Taiwan and to investigate the associated factors.

## Methods

### Study Design and Sampling

This study was based on a nationwide population-based cross-sectional survey, with participants enrolled between December 2011 and March 2013. To achieve a nationally representative sample, residents aged 65 and above in all 19 counties or cities across the country were identified as our target population. According to the 2010 census, there were 2,431,276 people aged 65 years and above among the entire population of 23 million in Taiwan. Computerized multistage random sampling from census data was performed. At the first stage, every official administrative district (“Li”) in each county was given a random number. In each county, the population of “Li”s was then sequentially summed by the random number order until 2% of the people aged 65 and above were obtained to create a list of sampling “Li”s. Because the population in each “Li” of every county is different, more “Li”s were sampled in some counties than in others. Initially, we sampled a total of 48,625 people. Among these people from each selected “Li”, approximately 60% were then further sampled at random to compile an address list with a total of 28,600 potential enrollees. We aimed to recruit 12,500 participants (0.5% among the older people) under the assumption of a participation rate of approximately 40%. With the assistance of the Ministry of Health and Welfare of Taiwan and local city governments, our study team obtained the address lists needed to perform a door-to-door survey.

### Standard Protocol Approvals and Patient Consents

This study was approved by the ethics committee at the National Taiwan University Hospital. Written informed consent was obtained from all participants or their proxies in the study.

### Training of Interviewers, Home Visit Procedure, and Quality Control

The field interviewers were primarily home care nurses. All interviewers participated in a 2-day training course. A neurologist presented lectures addressing the basic knowledge and diagnosis of dementia, and a clinical psychologist demonstrated the cognitive measurement tools. The lectures were followed by a 2-day internship at a medical center. Thus, all interviews were conducted by well-trained field interviewers with continuous quality control to achieve necessary quality standards. The inter-rater reliability of global CDR was substantial, with a kappa value of 0.671.

Participants were recruited after providing informed consent. An in-person interview was then performed to take a brief history related to cognitive and functional status, followed by a structured questionnaire with demographic data and mental tests. The interview process was performed according to an operational manual that defines all the variables examined in this questionnaire. Logic checks for inconsistency and auditing were performed by experienced supervisors to ensure the quality and reliability of the entered data. Difficult cases were examined and discussed by a consultant panel consisting of 4 neurologists and 1 clinical psychologist specializing in the diagnosis and care of people with dementia.

### Measurements for Cognitive and Functional Status

Objective mental evaluations included the Clinical Dementia Rating Scale (CDR) and the Taiwanese Mental State Examination (TMSE) [9], [10]. Normal TMSE results were defined as a score >24 in literate elders and >13 in illiterate elders [9], [10]. Functional status was assessed using the activities of daily living (ADL) scale for self-care activities such as washing, dressing, grooming, toileting, and eating and the instrumental activities of daily living (IADL) scale for activities such as cooking, shopping, laundry, and maintaining household finances.

### Diagnostic Criteria

The diagnosis for all-cause dementia was based on the core clinical criteria recommended by the National Institute on Aging-Alzheimer’s Association (NIA-AA) [11]. Cognitive status was determined from an in-person evaluation. A brief medical history was taken from the participant and a knowledgeable informant (who was a relative and a principal caregiver providing at least 10 hours of weekly direct care for the dementia patient). The medical history was taken to detect any insidious change of behavior or personality and any mental decline from previous levels of functioning and to determine whether this decline interfered with the ability to function at work or in routine activities. The cognitive impairment was not explained by delirium and major psychiatric disorders. In addition to the history of cognitive status, objective assessments including the CDR and TMSE were performed to evaluate memory, executive function, visual spatial ability, and language function. The severity of dementia was then determined by the CDR [7], [8]. Participants who were compatible with the NIA-AA criteria for all-cause dementia with a CDR score of 0.5 were defined as VMD [7], [8]. People with VMD had mild impairment in 2 or more cognitive domains as well as a mild decline in daily functions, whereby the cognitive deficits were sufficient to interfere with independence of daily living function as a result of abnormality in community affairs or at-home hobbies or as a result of personal care as assessed by the CDR. MCI was diagnosed, based on the criteria recommended by the NIA-AA, as a change in cognition with impairment in 1 or more cognitive domains but no evidence of impairment in social or occupational functioning as assessed by the CDR, ADL, and IADL [12]. For the diagnosis of non-dementia, individual should have none of the conditions listed in the NIA-AA core clinical criteria for all-cause dementia and have a CDR score of 0 as well as an education-adjusted TMSE within normal limits. Individual who did not fully meet the aforementioned criteria and whose diagnosis remained unclear after a discussion by the consultant panel were categorized in the unclassified group. For example, certain older people with major depression or another mental disorder or other serious physical illness might have impaired daily living function or impaired cognitive performance; consequently, their CDR score was > 0. This situation did not fully meet the NIA-AA criteria and was also not compatible with our definition of non-dementia.

### Statistical Analyses

Prevalence estimates were calculated as the number and percentage of cases with MCI or all-cause dementia in the total study sample. We estimated the overall and specific prevalence by gender and for each 5-year age group. The effects of age (5-year age categories), gender, and years of education on the prevalence of all-cause dementia and MCI were assessed with multiple logistic regression models to obtain odds ratios (ORs) and 95% confidence intervals (CIs) adjusted by possible confounding factors including smoking, drinking, habitual exercise, and comorbidities such as hypertension, diabetes mellitus, and cerebrovascular diseases. We performed all analyses using SAS statistical software (version 9.3 for Windows) with 2-tailed statistical tests.

## Results

**Figure 1:**
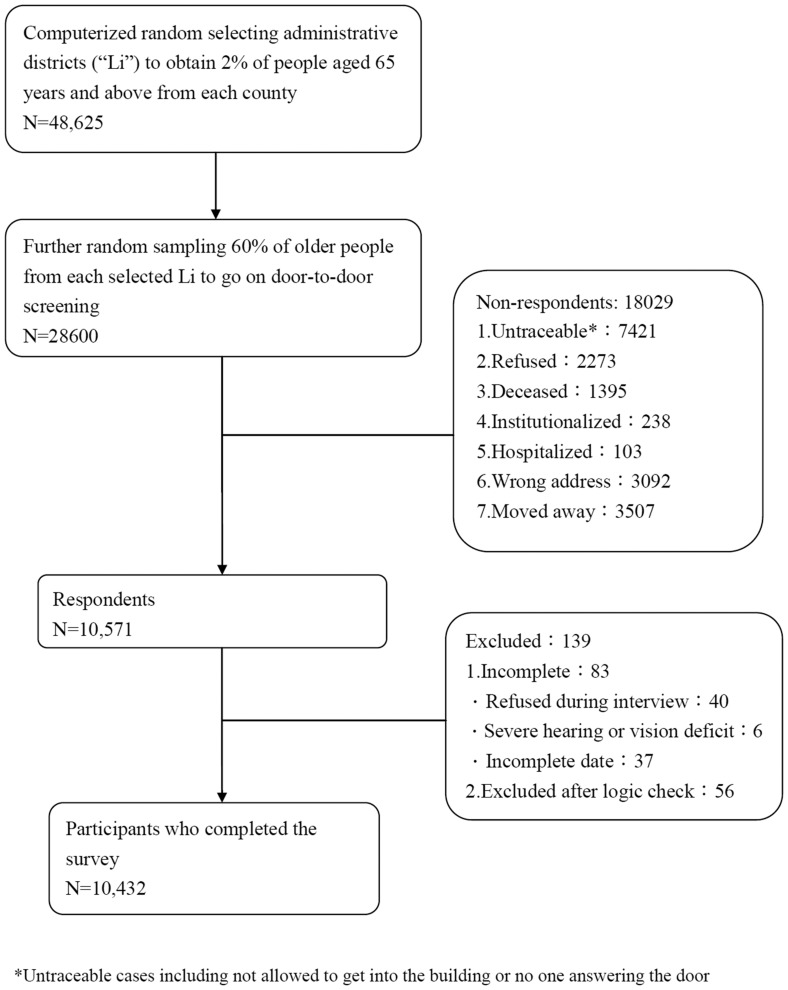
Flow chart of the enrollment of study participants.

Of the 28,600 subjects screened, 18,029 were non-respondents, primarily for the following reasons: interviewers were not permitted to enter subject’s residence, incorrect addresses were provided, subjects moved away from the listed address, or subjects refused to participate (Fig. 1). Of the 10,571 respondents, 139 were excluded due to incomplete or erroneous data (Fig. 1). We finally enrolled 10,432 subjects, reflecting a total participation rate of 36.5%. To check the representativeness of the participants of this study, we compared the general data of the respondents and non-respondents of Taipei city, the capital city of Taiwan, and those of Taoyuan county. Taipei city represents a highly urbanized area, whereas Taoyuan includes more suburbs and rural areas. The mean ages of respondents and non-respondents in Taipei city and Taoyuan were 75.2±7.2 years and 75.2±7.3 years, respectively (*p* = 0.83), and the proportions of female respondents and non-respondents were 51.7% and 51.1%, respectively (*p* = 0.19). There was no significant difference between the non-respondents and our study participants.

Table 1 shows the demographic characteristics of the participants. The mean age was 76.2±6.7 years, and 52.3% were women. A total of 3,351 participants (32.1%) were uneducated, and 4,719 (45.2%) had 6 years or less of education. The percentage of uneducated women (n = 2,589, 47.4%) was significantly higher than that of men (n = 762, 15.3%), and the percentage of participants with a higher education (college or university) was significantly less among women than among men (2.7% vs. 11.1%, *p*<0.0001).

**Table 1:** Demographic characteristics of study participants stratified by gender.

Variables*	All		Men		Women	
	(n = 10432)		(n = 4974)		(n = 5458, 52.3%)	
	n	%	n	%	n	%
Age						
65–<75 y/o	4798	46.0	2180	43.8	2618	48.0
75–<85	4323	41.4	2111	42.4	2212	40.5
> = 85	1311	12.6	683	13.7	628	11.5
Mean age (±SD)	76.2	6.7	76.5	6.7	75.9	6.7
Education Year						
0	3351	32.1	762	15.3	2589	47.4
< = 6	4719	45.2	2622	52.7	2097	38.4
6–12	1660	15.9	1039	20.9	622	11.4
>12	700	6.7	551	11.1	149	2.7
Lifestyle habits						
Smoking	2004	19.2	1906	38.3	98	1.8
Drinking	1261	12.1	1159	23.3	102	2.0
Regular exercise	4119	39.5	2161	43.5	1958	35.9
Comorbidities						
Hypertension	5338	51.2	2413	48.5	2925	53.6
Diabetes mellitus	2248	21.6	1000	20.1	1248	22.9
Cerebrovascular diseases	694	6.7	384	7.7	310	5.7

*Inconsistency between total population and population summed for individual variable was due to missing data.

Among the 10,432 participants, we identified 929 (8.91%) cases with dementia, including 366 (3.51%) with VMD with a CDR score of 0.5. There were 2,049 (19.63%) people suspected to have MCI, and 419 (4.02%) participants were categorized in the unclassified group. Table 2 shows that the age-adjusted prevalence of MCI was 18.76% (95% CI 17.91–19.61), with a peak of 26.24% at age 85–89 years and a decrease to 22.60% after 90 years of age. The age-adjusted prevalence of dementia (including VMD and a CDR
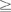
1) at ages 65–69, 70–74, 75–79, 80–84, 85–89, and 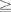;90 years was 3.40, 3.46, 7.19, 13.03, 21.92, and 36.88%, respectively; the prevalence approximately doubled for every 5-year increase in age after 70 years. The age-adjusted prevalence of all-cause dementia was 8.04% (95% CI 7.47–8.61), including a 3.25% (95% CI 2.89–3.61) prevalence of VMD. The prevalence of MCI was 18.76% (95% CI 17.91–19.61). We further computed the age-gender-adjusted prevalence, which was very similar to the aforementioned age-adjusted prevalence. The data showed an 18.78% (95% CI 18.03–19.53) prevalence of MCI and an 8.13% (95% CI 7.61–8.66) prevalence of dementia, including a 3.29% (95% CI 2.95–3.63) prevalence of VMD and a 4.84% (95% CI 4.43–5.26) prevalence of dementia with a CDR
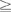
1 (Table 2).

**Table 2:** Age- and gender-specific prevalence of mild cognitive impairment (MCI) and all-cause dementia including very mild dementia (VMD).

Both sex		MCI		VMD		Dementia with CDR > = 1		All Dementia (VMD plus Dementia with CDR > = 1)	
		(n = 2049 )		(n = 366 )		(n = 563 )		(n = 929 )	
Age	n	Prevalence	95%CI	Prevalence	95%CI	Prevalence	95%CI	Prevalence	95%CI
65–69	1853	13.92	12.28–15.73	1.89	1.32–2.63	1.51	1.00–2.18	3.40	2.61–4.35
70–74	2945	16.43	15.00–17.97	1.73	1.29–2.28	1.73	1.29–2.28	3.46	2.82–4.20
75–79	2474	21.95	20.14–23.87	3.27	2.60–4.07	3.92	3.18–4.78	7.19	6.18–8.33
80–84	1849	23.47	21.32–25.79	4.65	3.72–5.74	8.38	7.12–9.81	13.03	11.44–14.79
85–89	926	26.24	23.05–29.76	7.56	5.89–9.55	14.36	12.03–17.02	21.92	19.01–25.15
> = 90	385	22.60	18.10–27.87	11.17	8.08–15.04	25.71	20.90–31.31	36.88	31.07–43.47
Total	10432	19.64	18.80–20.51	3.51	3.16–3.89	5.40	4.96–5.86	8.91	8.34–9.50
Age-adjusted prevalence (%)*		18.76	17.91–19.61	3.25	2.89–3.61	4.79	4.35–5.24	8.04	7.47–8.61
Age-gender-adjusted prevalence (%)*		18.78	18.03–19.53	3.29	2.95–3.63	4.84	4.43–5.26	8.13	7.61–8.66
**Men**		**MCI**		**VMD**		**Dementia with CDR > = 1**		**All Dementia (VMD plus Dementia with CDR > = 1)**	
		**(n = 827 )**		**(n = 130 )**		**(n = 220 )**		**(n = 350 )**	
**Age**	**n**	**Prevalence**	**95%CI**	**Prevalence**	**95%CI**	**Prevalence**	**95%CI**	**Prevalence**	**95%CI**
65–69	829	10.74	8.62–13.21	2.17	1.29–3.43	1.57	0.83–2.68	3.74	2.54–5.31
70–74	1351	13.18	11.31–15.26	1.04	0.57–1.74	1.33	0.79–2.11	2.37	1.62–3.34
75–79	1175	17.45	15.14–20.01	2.04	1.31–3.04	3.32	2.36–4.54	5.36	4.12–6.86
80–84	936	20.62	17.81–23.74	3.85	2.69–5.32	6.52	4.99–8.37	10.36	8.40–12.64
85–89	501	23.75	19.68–28.42	5.79	3.88–8.31	9.78	7.24–12.93	15.57	12.31–19.43
> = 90	182	23.63	17.10–31.82	4.95	2.26–9.39	21.98	15.70–29.93	26.92	19.92–35.59
Total	4974	16.63	15.51–17.80	2.61	2.18–3.10	4.42	3.86–5.05	7.04	6.32–7.81
Age-adjusted prevalence (%)*		15.57	14.44–16.70	2.49	2.04–2.94	3.87	3.29–4.46	6.36	5.62–7.10
**Women**		**MCI**		**VMD**		**Dementia with CDR > = 1**		**All Dementia (VMD plus Dementia with CDR > = 1)**	
		**(n = 1222 )**		**(n = 236 )**		**(n = 342 )**		**(n = 578 )**	
**Age**	**n**	**Prevalence**	**95%CI**	**Prevalence**	**95%CI**	**Prevalence**	**95%CI**	**Prevalence**	**95%CI**
65–69	1024	16.50	14.11–19.19	1.66	0.97–2.66	1.46	0.82–2.42	3.13	2.14–4.41
70–74	1594	19.20	17.11–21.47	2.32	1.63–3.20	2.07	1.43–2.91	4.39	3.42–5.55
75–79	1299	26.02	23.32–28.95	4.39	3.32–5.69	4.46	3.39–5.77	8.85	7.31–10.63
80–84	913	26.40	23.17–29.95	5.48	4.06–7.22	10.30	8.32–12.60	15.77	13.30–18.57
85–89	425	29.18	24.27–34.79	9.65	6.92–13.09	19.76	15.77–24.47	29.41	24.48–35.04
> = 90	203	21.67	15.75–29.10	16.75	11.60–23.40	29.06	22.12–37.49	45.81	36.98–56.12
Total	5458	22.39	21.15–23.68	4.32	3.79–4.91	6.28	5.64–6.99	10.61	9.76–11.51
Age-adjusted prevalence (%)*		21.63	20.38–22.89	4.00	3.45–4.56	5.71	5.05–6.38	9.71	8.85–10.58

*Age-adjusted prevalence was calculated with the use of Taiwan census data in 2012.

**Figure 2:**
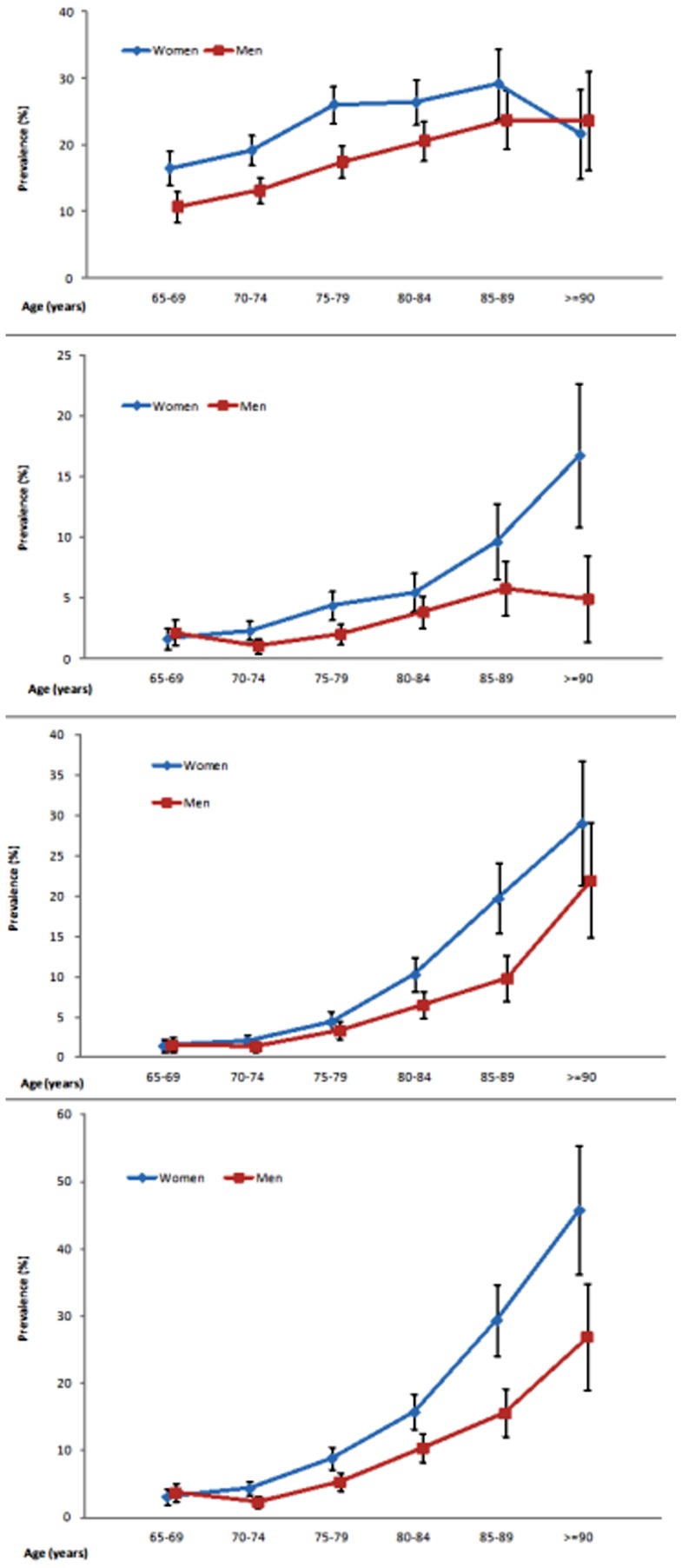
Age- and gender-specific prevalence and 95%CI of (A) mild cognitive impairment; (B) very mild dementia; (C) dementia with CDR> = 1; (D) all-cause dementia.

In terms of the differences between genders, the prevalence of MCI in women (21.63%, 95% CI 20.38–22.89) was higher than that in men (15.57%, 95% CI 14.44–16.70) (*p*<0.0001). The prevalence of all-cause dementia was also higher in women (9.71% vs. 6.36%, *p*<0.0001), including a higher prevalence of VMD (4.00% vs. 2.49%, *p*<0.0001). The age-specific prevalence of all-cause dementia approximately doubled for every 5-year increase after the age of 70 years in both women and men. The results also showed that the prevalence of MCI in women decreased significantly from 29.18% at age 86–89 years to 21.67% at age 90 or above, whereas the prevalence of MCI in men of both age groups was relatively stable (23.75% and 23.63%, respectively). In contrast to the decreased prevalence of MCI at the oldest age, the prevalence of VMD in women continued to increase from 9.65% at age 86–89 to a peak of 16.75% at the age of 90 or above. Unlike these prevalence changes in women, the prevalence of VMD in men aged 85–89 and that in men aged 90 or above was similar: 5.79% and 4.95%, respectively. Figure 2 shows the age- and gender-specific prevalence rates and 95% CIs for MCI, VMD, dementia with a CDR
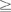
1 and all dementia.

Taiwan is primarily mountainous in the east, with gently sloping plains in the west, and is officially divided into 4 areas. The age-adjusted prevalence of dementia in the northern, central, southern, and eastern areas was 6.56% (95% CI 5.74–7.39), 12.28% (95% CI 11.06–13.49), 6.54% (95% CI 5.77–7.30) and 14.16% (95% CI 8.73–19.6), respectively, with significant differences among areas (*p*<0.001). The prevalence of MCI was also highest in the eastern area, followed by the central, northern, and southern areas (Table 3).

**Table 3:** Age-adjusted prevalence of MCI, VMD and total all-cause dementia in north, central, south and east areas of Taiwan.

	North		Central		South		East	
	(n = 3487)		(n = 2794)		(n = 3993)		(n = 158)	
	Prevalence	95%CI	Prevalence	95%CI	Prevalence	95%CI	Prevalence	95%CI
MCI	19.94	18.62–21.27	22.14	20.6–23.68	14.66	13.56–15.75	37.66	29.83–45.5
VMD	1.96	1.5–2.42	5.85	4.98–6.72	2.68	2.18–3.18	5.32	1.22–9.42
Dementia with CDR> = 1	4.60	3.91–5.3	6.43	5.52–7.34	3.85	3.26–4.45	8.84	3.51–14.17
Total dementia	6.56	5.74–7.39	12.28	11.06–13.49	6.54	5.77–7.30	14.16	8.73–19.6

*Age-adjusted prevalence was calculated with the use of Taiwan census data in 2012.

Table 4 presents the evaluation of the effects of age, gender, and education on MCI and dementia using multiple logistic regression after adjustment for other factors, including smoking, drinking, exercise, hypertension, diabetes mellitus, and cerebrovascular diseases. Compared with people aged 65–74, the OR of dementia was 3.28 (95% CI 2.68–4.02) in people aged 75–84 and increased to 16.38 for those aged 85 years or above. Compared with people who had received more than 12 years of education, the OR for dementia was 2.70 (95% CI 1.76–4.14) in those who had never been educated, with an OR of 2.91 (95% CI 1.30–6.52) in women and an OR of 2.08 (95% CI 1.24–3.50) in men (Table 4). Similarly, the education effect was significant for the risk of MCI and was especially high for women without any formal education, whose OR was 7.60 (95% CI 3.78–15.29) relative to those with a high educational level. After adjustment by age, education and the aforementioned associated factors, women were still at a higher risk of dementia than men, with an OR of 1.33 (95% CI 1.09–1.62, *p*<0.001) for dementia and an OR of 1.28 (95% CI 1.13–1.45, *p*<0.001) for MCI (Table 4). In addition, the *p* values for trend analyses on the variables of education and age were significant (all *p*<0.001), indicating the lower education or older age, the higher risk of dementia or MCI developed (Table 4).

**Table 4:** Estimated ORs for prevalent all-cause dementia and mild cognitive impairment (MCI) with stratification by gender*.

Dementia		All			Men			Women		
Variables		OR	95% CI	*p* for trend	OR	95% CI	*p* for trend	OR	95% CI	*p* for trend
Age				<0.001‡			<0.001‡			<0.001‡
	65–74	1.00			1.00			1.00		
	75–84	3.28	2.68–4.02		3.24	2.35–4.47		3.30	2.55–4.28	
	> = 85	16.38	12.93–20.74		12.33	8.60–17.69		20.94	15.22–28.81	
Education year				<0.001‡			<0.001‡			<0.001‡
	>12	1.00			1.00			1.00		
	0	2.70	1.76–4.14		2.08	1.24–3.50		2.91	1.30–6.52	
	6	1.61	1.04–2.48		1.74	1.05–2.90		1.34	0.59–3.07	
	7–12	1.16	0.72–1.85		1.31	0.75–2.27		0.91	0.37–2.23	
Gender				<0.001						
	Men	1.00			–	–		–	–	
	Women	1.33	1.09–1.62		–	–		–	–	
**MCI**		**All**			**Men**			**Women**		
**Variables**		**OR**	**95% CI**	***p*** ** for trend**	**OR**	**95% CI**	***p*** ** for trend**	**OR**	**95% CI**	***p*** ** for trend**
Age				<0.001‡			<0.001‡			<0.001‡
	65–74	1.00			1.00			1.00		
	75–84	1.65	1.47–1.84		1.71	1.43–2.04		1.62	1.40–1.87	
	> = 85	3.27	2.76–3.87		3.24	2.55–4.11		3.41	2.66–4.37	
Education year				<0.001‡			<0.001‡			<0.001‡
	>12	1.00			1.00			1.00		
	0	3.20	2.45–4.19		2.21	1.63–3.01		7.60	3.78–15.29	
	6	1.63	1.24–2.13		1.45	1.07–1.96		3.24	1.60–6.58	
	7–12	1.11	0.83–1.49		0.98	0.70–1.38		2.22	1.06–4.63	
Gender				<0.001						
	Men	1.00			–	–		–	–	
	Women	1.28	1.13–1.45		–	–		–	–	

*Adjusted by age, gender, education, smoking, drinking, regular exercise, hypertension, diabetes mellitus and cerebrovascular diseases.

‡ Logistic regression for linear trend.

## Discussion

In the Taiwanese population aged 65 years and above, the age-adjusted prevalence of MCI was 18.76%, and the prevalence of all-cause dementia was 8.04%, including a 3.25% prevalence of VMD. The overall prevalence for MCI and for dementia was higher in women than in men. The prevalence of dementia or VMD doubled for every 5-year increase after age 70. Age, gender, and education were significantly associated with MCI and dementia.

Previous studies in Taiwan reported that the prevalence of dementia in adults aged over 65 ranged between 1.7% and 4.3% based on surveys of local urban or rural areas [3], [13]–[16]. The pooled prevalence between 1991 and 1994 was approximately 3.6% based on 11,208 study subjects [17]. Most of the surveys conducted during that period used the DSM-III-R criteria for dementia. This study, conducted almost 2 decades after previous reports, showed an increase in the overall prevalence of dementia to approximately 8.0%. In approximately the same period, the percentage of the Taiwanese population aged over 65 years increased from 6.8% in 1992 to 11.1% in 2012. With the increase in both the aged population and the prevalence of dementia, the estimated number of people with dementia in Taiwan in 2012 was approximately 4 times that in 1992, increasing from 50,970 (3.6%) to 208,012 (8.0%). Therefore, the large increase in the prevalence of dementia might be due to the aging of the population and the inclusion of very mild cases in the present study. The difference between the DSM-III-R diagnostic criteria applied 2 decades ago and the recently updated NIA-AA recommendations for all-cause dementia might contribute, in part, to this increase [13], [14]. According to Alzheimer’s Disease International [18], there were an estimated 35.6 million people with dementia worldwide in 2010, and this number nearly doubles globally every 20 years. The marked increase in the population with dementia has also been noted in other countries and will pose a significant global societal and economic challenge [19]–[23].

Reports for the past decade on Asian populations aged over 65 indicate that the prevalence of all-cause dementia was approximately 4.1–5.8% in China [24], [25]. 4.2–8.5% in Japan [25], [26], and 5.2–9.2% in South Korea [25], [27]. A recent systematic review and meta-analysis showed that the dementia prevalence in Taiwan was estimated to be 5.7% [19]. A study from the 10/66 Dementia Research Group showed that the prevalence of dementia varied between 5.6% and 11.7% in Latin America, India, and China [28], and the prevalence ranged from 6 to 10% according to studies in the US and several European countries [8], [29]–[31]. The dementia diagnoses in most of those studies were based on the DSM-IV [19], [25]–[27] or on study-specific protocols [28], [29]. As the NIA-AA criteria used in this study have only been recommended since 2011, they had not been adopted when the aforementioned surveys were conducted. Different diagnostic criteria and study designs might contribute to the variations in the epidemiological estimates.

One study in the US showed that 22.2% of people aged over 70 had MCI [32]. Another study reported a 16% prevalence of MCI in people aged 70–89 years [33]. The prevalence of MCI was 12.1% in a German study with subjects aged 50–80 years and was 12.7% in a meta-analysis of a population aged 60 years and older in China [34]. Despite differences in methodology or age distribution, most of the studies reported a prevalence of MCI or cognitive impairment-no dementia in the range of 11–20% among people aged 65 and above [32], [35]–[37].

Although the recognition of VMD has specific implications for clinical management, and although pharmacological treatment for VMD cases might be a good strategy for early intervention, few prevalence studies have been conducted [7], [8], [38]. In addition, there is no consensus regarding the diagnostic criteria for VMD, but most studies have defined very mild cases as those with a CDR score of 0.5 [7], [8], [38]. In Denmark, the prevalence of all-cause dementia was 7.1%, including a 2.8% prevalence of VMD [8]. Another study showed that 8.5% of Hong Kong Chinese individuals aged 70 years or older had VMD [7], which was much higher than our figure of 3.8% (age 70+). The VMD prevalence found in the present study was close to that found for Denmark. Estimates of the prevalence of VMD are substantially affected by the definition and diagnostic procedures used. The higher prevalence of dementia found by this study compared with previous reports might be due, in part, to the inclusion of these very mild cases.

Compared with the Alzheimer’s Disease International consensus estimates by WHO region in 2008 [39] and the dementia report of the WHO in 2012 [40], the age-specific prevalence of all-cause dementia in people aged 65–69 in the present study was higher than those of all the listed regions [39], [40]. We found that the percentage of age-specific population 65–69 years was much lower (17.8%) than that in the national census data (28.7%). In contrast, for the population aged above 70, the age-specific population distributions in our sampled participants were similar to those in the overall national data (sampled vs. national census: 34.3% vs. 36.9 for ages 70–74; 28.8% vs. 27.5% for ages 75–79; 21.6% vs. 20.4% for ages 80–84; 10.8% vs. 10.7% for ages 85–89; and 4.5% vs. 4.5% for ages 90+). In part, the low participation rate among subjects aged 65–69 years might have resulted because many normal people at this age group in Taiwan are still working or leaving their home for leisure-time activities. However, those with cognitive impairments have a higher probability of being assessed because they are jobless or face limitations upon going out and are, therefore, more likely to stay at home. As a result, the prevalence of dementia in this age group (65–69 years) might be overestimated.

Compared with the worldwide estimates from the 2012 WHO dementia report [40] and a recent systematic review in China [20], our data for the age-specific prevalence of all-cause dementia at ages over 69 years were very similar to the estimated values in other countries. Take Australia and China for examples, the prevalence estimates were as following (Taiwan vs. Australia vs. China): 3.5% vs. 4.5 vs. 4.8% for ages 70–74; 7.2% vs. 7.5% vs. 8.5% for ages 75–79; 13.0% vs. 12.5% vs. 14.6% for ages 80–84; 21.9% vs. 20.3% vs. 24.3% for ages 85–89; and 36.9% vs. 38.3% vs. 39.0% for ages 90+. In addition, the results of this study were also similar to the estimates in North America and Western Europe (age groups 70 and above) in the 2012 WHO report. These values reflect an approximate doubling of prevalence every 5 years. Despite the differences among diagnostic criteria in case ascertainment, there were still similarities in the age-specific prevalence of dementia between countries.

An interesting observation was the marked decrease in MCI prevalence and the increase of VMD to a maximum value in the oldest women. For men, in contrast, both the MCI and VMD prevalence remained stable as age increased from 85–89 to 90+. These findings suggested that the proportion of cognitive status transitions from MCI to mild dementia is greater in women than in men at a later age. We do not yet have a plausible explanation of this observation, and further research is needed.

Age, gender, and education were significant associated factors for dementia. Uneducated people have a higher prevalence of not only dementia but also MCI compared with those with a high educational level. These results suggest that education may be a potential protective factor [41]. Among the people aged 65 and above in Taiwan, the percentage of women without any formal education (47.4%) is much greater than that of men (15.3 %). This difference may contribute to the higher frequency of dementia in women found in this study. The educational effects for MCI were also more significant in women than in men. Because of the potential for MCI to develop into dementia, adequate and suitable education for women might significantly decrease the overall prevalence of dementia. Nevertheless, after adjustment for age, education, and other common associated factors, women in our study still had a higher risk of dementia and MCI than men. This result may implicate other gender-related biological or psychosocial factors in the pathogenesis of cognitive impairment.

This study has several strengths. First, it is the only large-scale, nationwide epidemiology study to date for both dementia and MCI in Taiwan. Most previous studies in Taiwan were performed in local rural or urban areas and might not be nationally representative. Second, all participants in this study underwent a detailed in-person assessment. The inability to maintain daily living activities and usual social or occupational functions is essential for the differential diagnosis of MCI and dementia. It is difficult to make a clinical diagnosis without a thorough evaluation of the subject. Third, this study identified the prevalence of VMD, as epidemiological reports about the early stage of dementia are still uncommon. With advances in treatment, it is critical to detect milder forms of dementia earlier to obtain the maximal benefits of intervention. Fourth, different definitions for MCI have resulted in a wide range of estimates in previous reports. The case determination for all-cause dementia and MCI in this study was based on the core clinical criteria from the NIA-AA [11], [12]. This study is a very early, if not the first, epidemiological field survey adopting these newly revised criteria, which are expected to be one of the principal diagnostic guidelines for most dementia research in the future; thus, future data will be comparable to those of the present study.

An important limitation of the study was the low participation rate in this national survey, especially for people aged 65–69 years. Although all of the study participants were randomly sampled and the age and gender distribution between non-respondents and participants in the 2 selected city/county areas showed no significant differences, there still remained a residual selection bias. The higher probability of staying home for people with cognitive impairments than for those normally at work might overestimate the prevalence of dementia in the age group of 65–69 years. Because permission to enter the residences of non-respondents was denied, it was difficult for our research staff to clarify the retirement status or the actual reason why the non-respondents were not staying home and to evaluate how this factor influenced our results. Nevertheless, age standardization was used to adjust the difference in the age distribution between the sampled study participants and the entire national population. Another limitation is that the cognitive function of all of the participants was assessed primarily by clinical history, TMSE, and CDR without a detailed psychiatric evaluation to exclude other major psychiatric disorders that may impair cognitive function and might be misdiagnosed as dementia; this limitation applies although each difficult case was examined and discussed by a consultant panel consisting of 4 neurologists and 1 clinical psychologist specializing in the diagnosis and care of people with dementia. The lack of further diagnostic procedures such as brain imaging and blood tests for dementia classification is an additional limitation of this study. Definitive diagnoses and classifications of dementia are usually lacking in field surveys such as our study.

In conclusion, this nationwide epidemiological study showed that MCI affects a considerable percentage of the population of Taiwan aged 65 and above and is more prevalent than dementia in Taiwan. The inclusion of very mild cases of dementia resulted in a higher prevalence than that previously reported. In addition to old age, female gender and a low educational level are significant risk factors for MCI, VMD, and all-cause dementia.

## References

[1] Alzheimer’s Disease International (2013) Available: http://www.alz.co.uk/research/statistics.

[2] Statistical Yearbook of Interior (2013) Available: http://sowfmoigovtw/stat/year/elisthtm.

[3] Fuh JL, Wang SJ (2008) Dementia in Taiwan: past, present, and future. Acta Neurol Taiwan 17: 153–161. 18975520

[4] Busse A, Hensel A, Guhne U, Angermeyer MC, Riedel-Heller SG (2006) Mild cognitive impairment: long-term course of four clinical subtypes. Neurology 67: 2176–2185. 17190940 10.1212/01.wnl.0000249117.23318.e1

[5] Das SK, Bose P, Biswas A, Dutt A, Banerjee TK, et al (2007) An epidemiologic study of mild cognitive impairment in Kolkata, India. Neurology 68: 2019–2026. 17548552 10.1212/01.wnl.0000264424.76759.e6

[6] Luck T, Riedel-Heller SG, Kaduszkiewicz H, Bickel H, Jessen F, et al (2007) Mild cognitive impairment in general practice: age-specific prevalence and correlate results from the German study on ageing, cognition and dementia in primary care patients (AgeCoDe). Dement Geriatr Cogn Disord 24: 307–316. 17848793 10.1159/000108099

[7] Lam LC, Tam CW, Lui VW, Chan WC, Chan SS, et al (2008) Prevalence of very mild and mild dementia in community-dwelling older Chinese people in Hong Kong. International psychogeriatrics / IPA 20: 135–148. 10.1017/S1041610207006199 17892609

[8] Andersen K, Lolk A, Nielsen H, Andersen J, Olsen C, et al (1997) Prevalence of very mild to severe dementia in Denmark. Acta neurologica Scandinavica 96: 82–87. 9272182 10.1111/j.1600-0404.1997.tb00244.x

[9] Shyu YI, Yip PK (2001) Factor structure and explanatory variables of the Mini-Mental State Examination (MMSE) for elderly persons in Taiwan. J Formos Med Assoc 100: 676–683. 11760373

[10] 郭乃文, 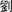 秀枝, 王珮芳, 徐道昌 (1989) 中文版「簡短式智能評估」(MMSE)之簡介. 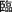 床醫學月刊 23: 39–42.

[11] McKhann GM, Knopman DS, Chertkow H, Hyman BT, Jack CR, et al (2011) The diagnosis of dementia due to Alzheimer’s disease: recommendations from the National Institute on Aging-Alzheimer’s Association workgroups on diagnostic guidelines for Alzheimer’s disease. Alzheimer’s & dementia : the journal of the Alzheimer’s Association 7: 263–269. 10.1016/j.jalz.2011.03.005 PMC3312024 21514250

[12] Albert MS, DeKosky ST, Dickson D, Dubois B, Feldman HH, et al (2011) The diagnosis of mild cognitive impairment due to Alzheimer’s disease: recommendations from the National Institute on Aging-Alzheimer’s Association workgroups on diagnostic guidelines for Alzheimer’s disease. Alzheimer’s & dementia : the journal of the Alzheimer’s Association 7: 270–279. 10.1016/j.jalz.2011.03.008 PMC3312027 21514249

[13] Liu HC, Lin KN, Teng EL, Wang SJ, Fuh JL, et al (1995) Prevalence and subtypes of dementia in Taiwan: a community survey of 5297 individuals. J Am Geriatr Soc 43: 144–149. 7836638 10.1111/j.1532-5415.1995.tb06379.x

[14] Liu CK, Lin RT, Chen YF, Tai CT, Yen YY, et al (1996) Prevalence of dementia in an urban area in Taiwan. J Formos Med Assoc 95: 762–768. 8961673

[15] Liu HC, Fuh JL, Wang SJ, Liu CY, Larson EB, et al (1998) Prevalence and subtypes of dementia in a rural Chinese population. Alzheimer Dis Assoc Disord 12: 127–134. 9772013 10.1097/00002093-199809000-00002

[16] Liu HC, Chou P, Lin KN, Wang SJ, Fuh JL, et al (1994) Assessing cognitive abilities and dementia in a predominantly illiterate population of older individuals in Kinmen. Psychological medicine 24: 763–770. 7991758 10.1017/s0033291700027914

[17] Liu CK TC, Lin RT, Lai CL (2007) Epidemiology of dementia in Taiwan. Research in Applied Psychology 7: 157–169.

[18] Dementia statistics ( 2013) Available: http://wwwalzcouk/research/statistics.

[19] Wu YT, Lee HY, Norton S, Chen C, Chen H, et al (2013) Prevalence studies of dementia in mainland china, Hong Kong and Taiwan: a systematic review and meta-analysis. PLoS One 8: e66252. 23776645 10.1371/journal.pone.0066252 PMC3679068

[20] Chan KY, Wang W, Wu JJ, Liu L, Theodoratou E, et al (2013) Epidemiology of Alzheimer’s disease and other forms of dementia in China, 1990–2010: a systematic review and analysis. Lancet 381: 2016–2023. 23746902 10.1016/S0140-6736(13)60221-4

[21] Hebert LE, Weuve J, Scherr PA, Evans DA (2013) Alzheimer disease in the United States (2010–2050) estimated using the 2010 census. Neurology 80: 1778–1783. 23390181 10.1212/WNL.0b013e31828726f5 PMC3719424

[22] Aggarwal NT, Tripathi M, Dodge HH, Alladi S, Anstey KJ (2012) Trends in Alzheimer’s disease and dementia in the Asian-Pacific region. Int J Alzheimers Dis 2012: 171327. 23304631 10.1155/2012/171327 PMC3523465

[23] Dodge HH, Buracchio TJ, Fisher GG, Kiyohara Y, Meguro K, et al (2012) Trends in the prevalence of dementia in Japan. Int J Alzheimers Dis 2012: 956354. 23091769 10.1155/2012/956354 PMC3469105

[24] Zhang Y, Xu Y, Nie H, Lei T, Wu Y, et al (2012) Prevalence of dementia and major dementia subtypes in the Chinese populations: a meta-analysis of dementia prevalence surveys, 1980–2010. J Clin Neurosci 19: 1333–1337. 22682650 10.1016/j.jocn.2012.01.029

[25] Catindig JA, Venketasubramanian N, Ikram MK, Chen C (2012) Epidemiology of dementia in Asia: insights on prevalence, trends and novel risk factors. J Neurol Sci 321: 11–16. 22877510 10.1016/j.jns.2012.07.023

[26] Ikeda M, Fukuhara R, Shigenobu K, Hokoishi K, Maki N, et al (2004) Dementia associated mental and behavioural disturbances in elderly people in the community: findings from the first Nakayama study. Journal of neurology, neurosurgery, and psychiatry 75: 146–148. PMC1757456 14707327

[27] Jhoo JH, Kim KW, Huh Y, Lee SB, Park JH, et al (2008) Prevalence of dementia and its subtypes in an elderly urban korean population: results from the Korean Longitudinal Study on Health And Aging (KLoSHA). Dement Geriatr Cogn Disord 26: 270–276. 18841012 10.1159/000160960

[28] Llibre Rodriguez JJ, Ferri CP, Acosta D, Guerra M, Huang Y, et al (2008) Prevalence of dementia in Latin America, India, and China: a population-based cross-sectional survey. Lancet 372: 464–474. 18657855 10.1016/S0140-6736(08)61002-8 PMC2854470

[29] Nowrangi MA, Rao V, Lyketsos CG (2011) Epidemiology, assessment, and treatment of dementia. Psychiatr Clin North Am 34: 275–294, vii. 21536159 10.1016/j.psc.2011.02.004

[30] Tognoni G, Ceravolo R, Nucciarone B, Bianchi F, Dell’Agnello G, et al (2005) From mild cognitive impairment to dementia: a prevalence study in a district of Tuscany, Italy. Acta neurologica Scandinavica 112: 65–71. 16008529 10.1111/j.1600-0404.2005.00444.x

[31] Kukull WA, Bowen JD (2002) Dementia epidemiology. Med Clin North Am 86 573–590. 12168560 10.1016/s0025-7125(02)00010-x

[32] Plassman BL, Langa KM, Fisher GG, Heeringa SG, Weir DR, et al (2008) Prevalence of cognitive impairment without dementia in the United States. Ann Intern Med 148: 427–434. 18347351 10.7326/0003-4819-148-6-200803180-00005 PMC2670458

[33] Petersen RC, Roberts RO, Knopman DS, Geda YE, Cha RH, et al (2010) Prevalence of mild cognitive impairment is higher in men. The Mayo Clinic Study of Aging. Neurology 75: 889–897. 20820000 10.1212/WNL.0b013e3181f11d85 PMC2938972

[34] Nie H, Xu Y, Liu B, Zhang Y, Lei T, et al (2011) The prevalence of mild cognitive impairment about elderly population in China: a meta-analysis. International journal of geriatric psychiatry 26: 558–563. 20878675 10.1002/gps.2579

[35] Larrieu S, Letenneur L, Orgogozo JM, Fabrigoule C, Amieva H, et al (2002) Incidence and outcome of mild cognitive impairment in a population-based prospective cohort. Neurology 59: 1594–1599. 12451203 10.1212/01.wnl.0000034176.07159.f8

[36] Ritchie K (2004) Mild cognitive impairment: an epidemiological perspective. Dialogues Clin Neurosci 6: 401–408. 22034212 10.31887/DCNS.2004.6.4/kritchie PMC3181815

[37] Gauthier S, Reisberg B, Zaudig M, Petersen RC, Ritchie K, et al (2006) Mild cognitive impairment. Lancet 367: 1262–1270. 16631882 10.1016/S0140-6736(06)68542-5

[38] Andersen K, Nielsen H, Lolk A, Andersen J, Becker I, et al (1999) Incidence of very mild to severe dementia and Alzheimer’s disease in Denmark: the Odense Study. Neurology 52: 85–90. 9921853 10.1212/wnl.52.1.85

[39] The prevalence of dementia worldwide. Alzheimer’s Disease International (2008) Available: http://www.alz.co.uk/adi/pdf/prevalence.pdf.

[40] WHO (2012) Dementia: a public health priority. Available: http://wwwwhoint/mental_health/publications/dementia_report_2012.

[41] Brayne C, Ince PG, Keage HA, McKeith IG, Matthews FE, et al (2010) Education, the brain and dementia: neuroprotection or compensation? Brain 133: 2210–2216. 20826429 10.1093/brain/awq185

